# Assessing osteopenia and osteoporosis with dual-energy x-ray absorptiometry studies in Fabry disease

**DOI:** 10.1186/s13023-025-03601-x

**Published:** 2025-04-30

**Authors:** Alyaa Shmara, Grace Lee, Mania Mgdsyan, Kathy Hall, Nadia Sadri, Angela Martin-Rios, Kelsey Valentine, Tatiana Kain, Madeleine Pahl, Lynda E. Polgreen, Virginia Kimonis

**Affiliations:** 1 Division of Genetics and Genomic Medicine, Department of Pediatrics, University of California Irvine, 101 The City Drive South, ZC4482, Orange, CA 92868 USA; 2 College of Osteopathic Medicine, Western University of Health Sciences, Pomona, CA USA; 3 College of Arts and Sciences, New York University, New York, NY USA; 4 Nuclear Medicine, Department of Radiology, University of California, Irvine, CA USA; 5 Division of Nephrology, Department of Medicine, Hypertension, and Kidney Transplantation, University of California, Irvine, CA USA; 6 The Lundquist Institute for Biomedical Innovation at Harbor, UCLA Medical Center, Torrance, CA USA; 7 Department of Neurology, University of California, Irvine, CA USA; 8 Department of Pathology, University of California, Irvine, CA USA

**Keywords:** Fabry disease, DXA, Bone mineral density, Enzyme replacement therapy

## Abstract

**Background:**

Fabry disease (FD) is a rare multi-systemic lysosomal storage disease that affects the heart and kidneys most significantly. An underappreciated manifestation of FD is reduced bone mineral density. Currently, there are no specific guidelines for routine bone density assessments, and treatment of osteoporosis and osteopenia in FD.

**Materials and methods:**

To ascertain the frequency of low bone mineral density in FD we studied dual-energy x-ray absorptiometry (DXA) scans obtained as part of routine care from a cohort of 25 individuals followed at the University of California—Irvine Medical Center for the period 2008–2023. The most recent BMD results for the lumbar spine and femoral neck were collected from 12 males and 13 females to examine the prevalence of low bone mineral density. The lowest Z- and/or T-scores of either lumbar spine or femoral neck were selected for analysis. Demographic factors, disease and ERT status, and other laboratory values were collected concurrently (within ± 9 months) with DXA scan results and were analyzed with Z- and T-scores to assess for correlations. In our cohort the mean age was 51 years (median 56 years, range 18–77 years). The Z-scores for all participants and T-scores from postmenopausal women and men ≥ 50-year-old were analyzed and correlated with various measures including disease duration, BMI, renal function (measured by eGFR), plasma GL3, Lyso-GL3, calcium, vitamin D, and alkaline phosphatase levels. These parameters were concurrent with DXA scan results.

**Results:**

The average Z-score for all the participants was −1.2 ± 1.3 (range −4.6 to 1.6). Twenty-four percent of all participants (*n = *6) had significantly low Z-scores ≤ −2.0. To identify the frequency of subjects with osteopenia, defined as T-score between −1.0 and −2.5 and osteoporosis defined as T-score < −2.5, T-scores were analyzed in postmenopausal women (*n = *8) and men 50 years and older (*n = *7). Of these 15 individuals, average T-score was −2.2 ± 1.3 (range −5.4 to 0.3), and 86.7% (*n = *13) had abnormal results (osteopenia and osteoporosis), 53.3% (*n = *8) had osteoporosis and 33.3% (*n = *5) had osteoporosis. We found a significant difference in Z-scores between male (−1.98 ± 1.33) and female patients (−.45 ± 0.82) t (23) = 3.487 (*p = * < 0.001). We did not find any differences in z-scores between different ethnic backgrounds. There was a strong negative correlation between Z-scores and Lyso-GL3 levels [r (15) = −.72, *p = *.001] and a moderate positive correlation between Z-scores and body mass index (BMI) [r (23) = .43, *p = *.033]. No correlation was found between Z-scores and calcium levels. There is a strong negative correlation between T-scores and Lyso-GL3 levels [r (8) = -.86, *p = *.001] and a negative correlation between T-scores and participants’ ages at the time of DXA [r (13) = −.57, *p = *0.028]. There is a positive correlation between T- scores and calcium levels [r (12) = .58, *p = *0.030]. No significant correlation was observed between T-scores and BMI. There was no correlation between Z or T- scores and disease duration, duration of ERT use, renal function (measured by eGFR), GL3, creatinine, alkaline phosphatase levels, or their use of vitamin D or concomitant antiepileptic medications.

**Conclusion:**

The findings of this cohort highlight the high prevalence of low bone mineral density in FD and correlations of low Z and T- scores with elevated levels of Lyso-GL3, and low calcium levels. We did not find correlations with renal function, and vitamin D levels. We discuss etiology, prevention, and treatment strategies for osteopenia/osteoporosis in Fabry disease.

**Supplementary Information:**

The online version contains supplementary material available at 10.1186/s13023-025-03601-x.

## Introduction

Fabry disease (FD) is a rare X-linked lysosomal storage disease caused by pathogenic variants in the *GLA* gene that lead to deficiency of the a-galactosidase A (AGAL-A) enzyme, which in turn leads to accumulation of globotriaosylceramide (GL-3) and globotriaosylphingosine (Lyso-GL3) in the lysosomes. Fabry disease is found among all ethnic, racial, and demographic groups. The incidence of classic FD has been projected form 1:50,000 to 1:117,000 males while late-onset FD is more common and affects about 1 in every 1,500 to 4,000 males [1]. The reported prevalence of FD detected through newborn screening per births is 1:7057 in Japan, 1:3100 in Italy, 1:3859 in Austria, and 1:1250 in Taiwan. In the US, the reported incidence is 1:8454 in Illinois (including the p.A143T variant), and 1:2913 in Missouri [2]. Individuals with FD are affected in a multi-systemic manner and require multi-disciplinary specialist care. The heart and kidneys are affected most significantly and can lead to organ failure. Other common symptoms of FD include, but are not limited to, peripheral neuropathy, corneal whorls, angiokeratomas, hearing loss, and hypohidrosis [1]. First reported in 2005, twenty hemizygous male patients with Fabry disease have been observed to have an increased risk of low bone mineral density (BMD), an underappreciated association [3]. In a cross-sectional study in 53 patients in Denmark at a mean age of 40 years with untreated FD, 46% (*n = *24) of the patients had femoral neck osteopenia and 27% (*n = *14) had osteopenia of the lumbar spine [4]. Another study in France retrospectively observed the musculoskeletal manifestations in a cohort of 14 Fabry patients who had undergone bone absorptiometry [5]. Fifty seven percent (*n = *8) had abnormal results. Of the three males with osteoporosis, two had vertebral fractures. Osteoporosis was diagnosed in three females while two females had osteopenia.

Possible causes of osteoporosis and osteopenia include reduced calcium absorption, low vitamin D levels, renal failure, secondary hyperparathyroidism, steroid therapy, and the use of antiepileptic agents for pain control. However, none of which have been clearly linked to FD as being the primary explanation [3–8]. Currently, there are no specific guidelines for routine bone density assessment and treatment of osteoporosis and osteopenia as part of FD standard of care. Moreover, despite being a subject of interest to FD researchers, there is paucity of related awareness information for patients in prominent rare disease and patient support sites. The aims of this retrospective study are to assess the prevalence of osteoporosis and osteopenia in FD patients; identify potential correlations between low BMD and demographic or clinical factors including disease duration, duration of ERT use, BMI, renal function (measured by eGFR), creatinine, GL3, Lyso-GL3, calcium, vitamin D, and alkaline phosphatase levels; and to provide insight on the effect of FD treatment on bone density.

## Methods

The study cohort consisted of 25 individuals (12 males and 13 females) at a mean age of 51.1 ± 14.4 years, (median age 56 years, range 18–77 years) and a confirmed clinical and molecular diagnosis of FD (Table 1). This retrospective study was conducted over the course of 2008- 2023. Patients provided written informed consent for the IRB approved study at the University of California, Irvine (HS#2008–6631). Clinical data was collected in an outpatient multidisciplinary clinic where they received their care for FD and outside medical records. Dual-energy x-ray absorptiometry (DXA) scans were recommended for all patients for BMD assessment at annual intervals [9–13]. Of special note is the variability in the DXA scans because not all scans from this cohort were performed at our sites at UC Irvine (Supplemental Table 1). The most recent BMD results for the lumbar spine and femoral neck were collected and the lowest Z- and/or T- scores of either lumbar spine or femoral neck were selected for analysis. Demographic factors, disease and ERT status, and other laboratory values were obtained concurrently (within ± 9 months of DXA scans) and were analyzed with Z- and T-scores to assess for correlations.

**Table 1:** Summary of demographics and clinical parameters of 25 patients with Fabry disease

Parameter	N	Minimum	Maximum	Mean	Median	Standard deviation
Age at time of DXA (years)	25	18	77	51.12	56	14.40
Age at diagnosis (years)	25	12	77	39.84	37	17.81
Duration of disease (years)	25	0	42	11.48	9	9.99
Age of ERT initiation (years)	22	15	77	43	45	17.89
BMI (kg /m^2^)	25	16.3	35.61	24.69	25.09	4.93
Creatinine (mg/dL)	23	0.61	7.58	1.27	0.91	1.42
Calcium (mg/dL)	22	7.9	10	9.27	9.35	0.51
T-score	18	−5.4	0.3	−2.14	−2	1.28
Z−score	25	−4.6	1.6	−1.19	−0.9	1.33
Lyso GL3 (ng/mL)	17	1.4	23	6.82	6.7	5.61
GL3 (mcg/mL)	22	0	5	2.78	2.88	1.41
25−Hydroxy vitamin D (ng/mL)	11	14	71	40.45	35	16.73
Alkaline phosphatase (IU/L)	23	45	158	76.57	74	25.24
eGFR (mL/min) CKD−EPI 2021	23	6.8	142.8	82.38	82.3	28.06

Z−score is a calculated value of BMD in the patient when compared to individuals of the same age group, sex, and race (NHANES III database) [14]. We first analyzed Z−scores for the entire cohort to explore if patients with FD have lower BMD than expected for their age, sex, and race. Subsequently, to determine the frequency of osteoporosis and osteopenia, T−scores were used to define bone density status in postmenopausal women and men aged 50 years and older: a T-score of −1 to −2.5 indicates osteopenia. A T-score of −2.5 or less indicates osteoporosis [7]. A T-score is a calculated value of BMD in the patient when compared to a healthy 20–29-year-old white, female, reference population [14].

For this study we collected data on serum GL-3, lysosomal-GL3 (lyso-GL3) [6], calcium, 25-hydroxy vitamin D, alkaline phosphatase, serum creatinine, and estimated glomerular filtration rate (eGFR) using CKD-EPI (2021 update) equation defined as: GFR = 141 * min (Scr/κ,1) α * max (Scr/κ, 1)−1.209 * 0.993Age * 1.018 [if female] * 1.159 [if black]. Scr is serum creatinine (mg/dL), κ is 0.7 for females and 0.9 for males, α is −0.329 for females and −0.411 for males, min indicates the minimum of Scr/κ or 1, and max indicates the maximum of Scr/κ or 1[15]. We also collected available data on BMI, duration of FD, Enzyme Replacement Therapy (ERT) use, and the use of steroids and antiepileptic medications. Due to the retrospective nature of the study, clinical and laboratory data were collected whenever available and concurrent (within ± 9 months) with Z- and T-scores (Table 1 and Supplemental Table 1).

Statistical analysis was performed using student's t-tests and ANOVA to calculate differences between data reported as categorical and continuous variables, such as ethnicity and T-score. Analysis using Pearson’s correlation was used to calculate correlations between continuous data and correlation coefficient was computed to assess the linear relationship between variables such as T-scores and Lyso-GL3. Statistical analysis was performed using SPSS (IBM Corp. Released 2020. IBM SPSS Statistics for Windows, Version 29.0 Armonk, NY: IBM Corp). A chi-square test of independence was used to evaluate the relationship between the participants’ use of antiepileptic medications or vitamin D and their Z or T-scores. Statistical significance using the Pearson χ^2^ test for categorical variables was demonstrated when a p value of < 0.05 was obtained.

## Results

### Participant demographics

The study population consisted of 12 males and 13 females at a median age of 56 years (range 18–77 years). The average age of diagnosis among participants was 39.84 ± 17.81 years of age. Of the 25 individuals in this cohort, 22 (88%) were on ERT, with an average ERT initiation age of 42.5 ± 17.9 years (range 15–77 years) (Table 1). The largest portion of this cohort self-identified as Caucasian (40%) and Hispanic (40%), while (20%) identified as Asian (Supplemental Table 1).

This cohort of 25 patients represented 17 families each with unique genotypes, and 4 families had multiple family members. There were no obvious genotype/ phenotype correlations (Supplemental Table 1).

**Fig. 1:**
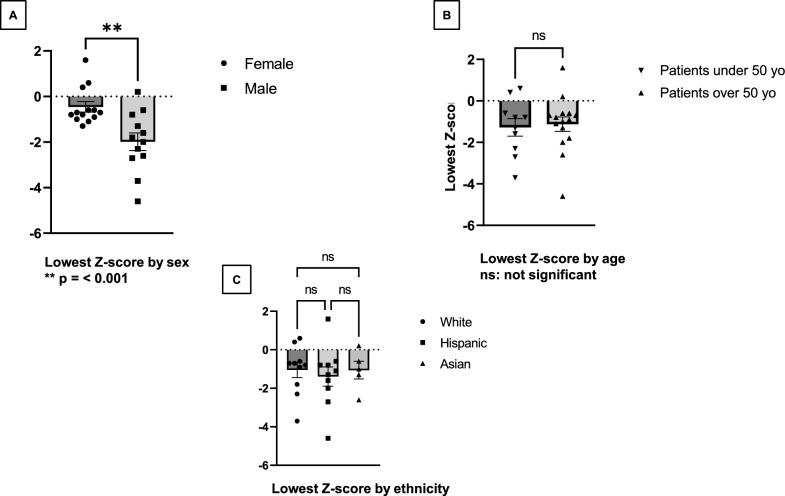
Differences in Z-scores by sex, age, and ethnicity

We found a significant difference in Z-scores between male (M = −1.98, SD = 1.33) and female patients (M = −0.45, SD = 0.82) t (23) = 3.487, *p = * < 0.001 with lower Z-scores in males compared to females (Fig. 1A). There was no difference in Z-scores between participants above and under age 50 years (Fig. 1B). We did not find any differences in Z-scores between different ethnic backgrounds (Fig. 1C). In our cohort, there are eight individuals with chronic kidney disease (CKD) stage 1, thirteen individuals with CKD stage 2, one individual with CKD stage 3b, and one individual with CKD stage 5 [15] (Supplemental Table 1).

Five individuals (20%) were taking antiepileptic medications (including gabapentinoids), two of whom had normal BMD (Supplemental Table 1). Chi-square test to evaluate the relationship between Z- or T- scores and the use of antiepileptic medications was not significant. Z- score χ2 ([1], *N = *[2]) = [0.877], *p = *[0.349]. T- score χ2 ([1], *N = *[2]) = [0.195], *p = *[0.659].

Two individuals started taking bisphosphonate after being diagnosed with osteoporosis. Notably, one patient (Participant 14 in Supplemental Table 1) developed a severe reaction to zoledronic acid which was discontinued, and he was later treated with alendronate. Fifteen individuals (60%) were taking vitamin D (Supplemental Table 1). Chi-square test to evaluate the relationship between Z- or T- scores and the use of vitamin D was not significant. Z- score χ2 ([1], *N = *[2]) = [1.791], *p = *[0.181]. T- score χ2 ([1], *N = *[2]) = [1.068], *p = *[0.301]. There are 2 patients with systemic use of steroids (participants 15 and 23 in Supplemental Table 1).

One patient in our cohort (Participant 14 in Supplemental Table 1) who reported spending up to 40 h per week in a sensory deprivation floatation, had early-onset low BMD at the age of 36 years. Reviewing participants’ records revealed five individuals with history of fractures; two of whom were within 9 months of the time of DXA results (Supplemental Table 1).

### Dual-energy x-ray absorptiometry (DXA) scans

The average Z-score for all the participants was −1.2 ± 1.3 (range −4.6 to + 1.6). Twenty-four percent (*n = *6) of all participants had Z-score below the expected range for age (defined as ≤ −2.0). To identify the frequency of subjects with osteopenia, T-scores were analyzed in postmenopausal women (*n = *8) and men 50 years and older (*n = *7). Of 15 individuals with a reported T-score, 86.7% had abnormal results (osteopenia and osteoporosis), eight (53.3%) individuals had osteopenia, and five (33.3%) individuals had osteoporosis. Only two (13.3%) individuals were within normal limits. It is worth noting the DXA scan results of one of our patients, a 19-year-old male who began experiencing FD symptoms at 12 years old. His Z-score was −3.60 in the spine, and his femoral neck Z-score was calculated to be −2.7 (Participant ID 16 in Supplemental Table 1). His unusual results at his age are unique and an outlier in our dataset. He had a classic mutation and his Lyso GL3 was high at 11 ng/mL. The reference interval (< 0.30 ng/mL) was determined using plasma from 120 non-disease adults (26 female and 94 male).

### DXA correlations with clinical parameters of the patients

**Fig. 2:**
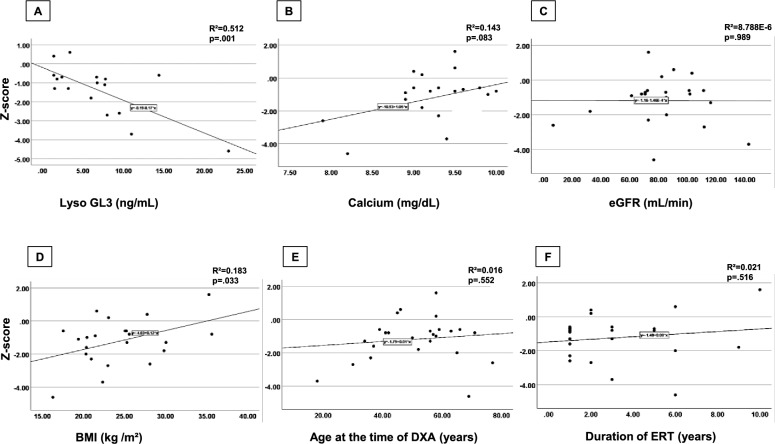
Correlation between Z-scores and clinical parameters in participants

There was a strong negative correlation between Z-scores of all participants and Lyso-GL3 levels [r (15) = −0.72, *p = *0.001] implicating that as the Lyso-GL3 levels increase, Z-scores decrease (Fig. 2A). There was a moderate positive correlation between Z-scores and body mass index (BMI) [r (23) = 0.43, *p = *0.033] which indicates that Z-score increased with higher BMI (Fig. 2D). No significant correlation was observed between Z-scores and serum calcium level [r (20) = 0.38, *p = *0.083] (Fig. 2B). Likewise, there was no significant correlation was observed between Z-scores and participants’ ages at the time of DXA [r (23) = 0.13, *p = *0.552] (Fig. 2E).

**Fig. 3:**
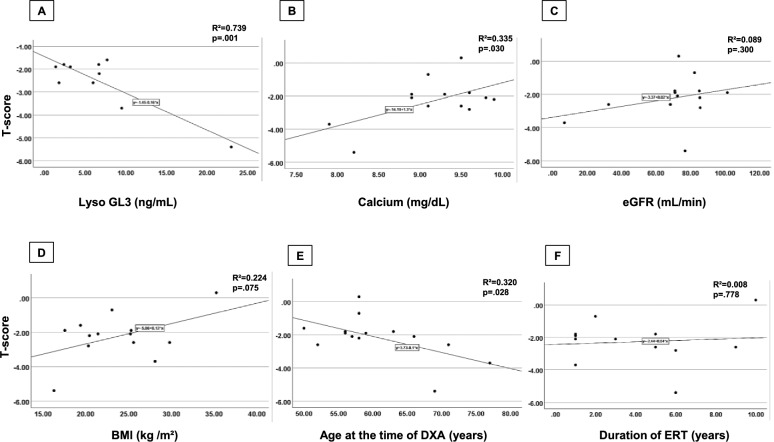
Correlation between T-scores and clinical parameters in postmenopausal females and males over age 50 years

There was a strong negative correlation between T-scores and Lyso-GL3 levels [r (8) = −0.86, *p = *0.001] (Fig. 3A). There was a moderate positive correlation between T- scores and calcium levels [r (12) = 0.58, *p = *0.030] suggesting that the higher calcium level, the higher the T-score. (Fig. 3B). There was a moderate negative correlation between T-scores and participants’ ages at the time of DXA [r (13) = −0.57, *p = *0.028] indicating that older individuals had lower T-scores. (Fig. 3E) No significant correlation was observed between T-scores and BMI [r (13) = 0.47, *p = *0.075].

No association with statistical significance was noted between Z- or T-scores and any of the following parameters: renal function (measured by eGFR) [r (21) = −0.00 *p = *0.989] and [r (12) = 0.30, *p = *0.300] respectively (Figs. 2C and 3C), disease duration [r (22) = 0.01 *p = *0.961] and [r (12) = −0.06, *p = *0.850] respectively, duration of ERT use [r (20) = 0.15 *p = *0.516] and [r (10) = 0.09, *p = *0.778] respectively (Figs. 2F and 3F), GL3 [r (17) = −0.41 *p = *0.080] and [r (10) = −0.06, *p = *0.861] respectively, creatinine [r (21) = −0.26 *p = *0.238] and [r (12) = −0.35, *p = *0.223] respectively, vitamin D [r (9) = −0.09 *p = *0.791] and [r (4) = 0.24, *p = *0.648] respectively, or alkaline phosphatase levels [r (21) = 0.02 *p = *0.925] and [r (12) = 0.49, *p = *0.078].

## Discussion

DXA scans are the standard tool for measuring BMD in people who may be at risk for bone loss, such as those of older age or those with a comorbidity such as FD. DXA has been shown to have excellent accuracy and precision with low exposure to radiation [9–13]. Studies have revealed a strong correlation between fracture risk and bone mechanical strength with BMD measured by DXA [12, 13]. Our results indicated there is a high frequency of low BMD indicated by low Z and T scores in FD. Twenty-four percent of all 25 participants had Z-score below the expected range (−2SD) for age. However, among the group of postmenopausal females and men > 50 years (*n = *15) 86.7% had abnormal T- score results (53.3% osteopenia, 33.3% osteoporosis). We found a significant difference between the sexes with males having a lower Z-scores compared to females.

Our data is consistent with a recent observational study of 15 patients with FD that found Lyso-GL3 levels to be significantly related to the lumbar spine and femoral BMD, and not surprisingly that males had lower Z-scores of the lumbar spine and femoral neck than females [8]. Available studies suggest that ERT aids to offset the effect of low BMD due to FD [16]. Overall, our results are consistent with current literature, suggesting there is an association between FD and BMD, and that the more severe the symptoms of FD, the more likely the association with osteopenia or osteoporosis. The strong negative correlation between Z-scores of participants and Lyso-GL3 levels suggests the role of optimal management of FD in preventing low BMD.

### Calcium and bone health

Our results suggest a statistically significant correlation between lower calcium levels and lower T-scores. In healthy individuals, plasma calcium levels are tightly regulated to optimize bone health. Parathyroid hormone (PTH), 1,25 dihydroxycholecalciferol (1,25 (OH)2D3) and calcitonin act on target tissues (i.e., kidney, bone, and intestine) to regulate absorption, excretion, secretion, and storage of calcium in bone [17]. There is supportive evidence that increased dietary calcium intake can reduce the risk of osteoporosis particularly in men over the age of 50 and postmenopausal women [17]. Recently, a correlation was identified between low hip BMD and high calciprotein particles (CPP-II) in patients with FD. CPP are colloidal mineral-protein complexes that form in extracellular fluid and facilitate the stabilization, transport, and clearance of excess minerals from the circulation. CPP exist in primary (CPP-I) and secondary (CPP-II) form, both of which are reported to be raised in pathological states [18]. One study that looked at mouse models of FD with impaired functioning of the medullary thick ascending limb of the nephron, observed that a decrease in plasma calcium induced an increase in PTH, leading to secondary hyperparathyroidism, which resulted in accelerated bone resorption [19]. We did not find a significant correlation between Z- or T-scores and vitamin D levels in our cohort. Nevertheless, the effect of serum calcium level on BMD in this cohort should be carefully interpreted in view of lack of related parameters including 1,25 (OH)2D3, serum PTH, and phosphate levels.

### BMI and bone health

Our data revealed a statistically significant correlation between BMI and Z-scores indicating that BMI can have protective effects on bone health in this patient population. However, understanding the relationship between BMI and BMD is complex because obesity has been shown to have protective as well as detrimental effects on bone health. Certain studies have shown a lower incidence of hip fractures in obese subjects, while other studies have shown a greater number of low-trauma fractures in overweight and obese subjects. Overall, DXA studies have shown that BMD is increased in obese individuals. However, this increase in BMD may not be significant enough to offer protection against the forces of falling [20].

### Kidney failure and bone health

Renal dysfunction is a known symptom of FD [21] and has been shown to be correlated to osteopenia and osteoporosis [22]. The current published data linking FD to low bone BMD, as well as the data examining the cause of this link is limited. Possible links between low BMD and FD include secondary hyperparathyroidism caused by renal dysfunction [21].

The correlation between GL-3 accumulation and pathogenic mechanism for renal damage has been documented in FD [21, 23]. Renal biopsies of Fabry patients often show signs of histological damage before proteinuria or elevated serum creatinine is detected. Accumulation of GL3 in the kidney results in cellular damage and irreversible organ damage because of fibrosis, apoptosis, and inflammation, eventually leading to renal dysfunction and end-stage renal disease. Treatment with ERT is effective in clearing GL3 and to slow renal decline [24].

The demonstrated high frequency of osteopenia and osteoporosis in our cohort, with absence of correlation between eGFR and creatinine to both Z and T score in these patients, suggest that the bone disease in this cohort is not caused by renal damage, but potentially by a different pathological condition secondary to FD. We hypothesize that in this cohort, Fabry disease is the direct cause of bone alterations as evidenced by the strong negative correlation between Lyso GL3 and both Z and T-scores. More studies should be developed to validate the findings of this study.

### Flotation therapy and pain control

Three of our patients utilize floatation therapy to alleviate pain and anxiety. The procedure involves submersion in water baths that are saturated with magnesium sulfate in a dark and soundless room. This is meant to restrict stimulation from gravity, sound, and light, and has shown to significantly reduce pain intensity, as one clinical trial with 37 participants noted [3, 25].

While this nonpharmacological method may provide temporary relief from neuropathic pain, it is important to note the potential consequence it may have on bone health, especially when used in Fabry patients who are already at risk for developing osteopenia and/or osteoporosis secondary to renal failure. One patient in our cohort with osteoporosis reported spending excessive time in a sensory deprivation flotation tank. While there were no prior DXA scans to measure the rate of bone loss in this individual, it is believed that the overuse of floatation therapy may have been contributory, since it has been well established that astronauts experience bone loss in response to the microgravity of space [26].

It has been suggested that antiepileptics usage for pain control to FD patients as being the primary explanation, but none have been clearly linked [7]. In our cohort, 5 individuals (20%) were taking antiepileptic medications, 2 of whom had normal BMD.

### Treatment of osteoporosis in Fabry patients with diminished renal function

It is essential to explore treatment options for osteoporosis in FD, considering that five patients in our cohort had a history of fractures, with two of these incidents occurring at the time of the DXA assessment. We found that fifteen individuals (60%) were taking vitamin D supplementation and only two patients were prescribed bisphosphonates for osteoporosis treatment even though bisphosphonates are a common treatment for osteoporosis. In the past the U.S. Food and Drug Administration (FDA) issued a warning regarding the use of certain osteoporosis medications in the setting of CKD, including bisphosphonates due to reduced renal clearance, and denosumab (Prolia) due to hypercalcemia [27]. Despite these precautions, bisphosphonates can be beneficial for many patients with osteoporosis and diminished renal function when used at a lower dose and infusion rate [22, 28] and so could be considered for treatment of osteoporosis in patients with Fabry disease and diminished renal function.

Our study has potential limitations. First, is the intermachine differences in DXA measurements. This is important to note, since not all DXA scans from this cohort were performed at our sites at UC Irvine. Site-based variations may occur due to using different machines, lack of adherence to manufacturers’ recommendations for system maintenance and quality control. Additionally, discrepancies in the training of technologists may yield inconsistent DXA results [13, 29]. This necessitates the need for future studies in FD patients using standardized DXA scan.

Other limitations include the relatively small number of participants and limited serum samples available for analysis which is attributed to the rarity of FD and the retrospective nature of the study. The effect of serum calcium level, kidney failure, or symptomatic treatment on BMD should be carefully interpreted in view of the limited choice of assessed parameters including 1,25 (OH)2D3, and the lack of serum PTH and phosphate levels.

## Conclusion

We undertook this study because limited data has been published on the effect of FD treatment on bone density. In this cohort of 25 patients with FD, we highlight the high prevalence of low bone mineral density and correlations between DXA scores and Lyso- GL3, BMI, and calcium.

This study reinforces the importance of treatment such as ERT which lowers the Lyso- GL3, and maintaining calcium levels with dietary intake of calcium and vitamin D. Better understanding of the effects of FD on BMD will lead to better standard of care in a truly multidisciplinary manner and early treatment in prevention of fractures related to low mineral density. Results from our studies demonstrate the need for DXA scans to be performed on patients at an earlier stage of the disease and included as routine care.

## Supplementary Information

Additional file 1

## Data Availability

The data that support the findings of this study are not openly available due to reasons of sensitivity and are available from the corresponding author upon reasonable request. Data are in controlled access data storage at the University of California, Irvine.
